# PCDq: human protein complex database with quality index which summarizes different levels of evidences of protein complexes predicted from H-Invitational protein-protein interactions integrative dataset

**DOI:** 10.1186/1752-0509-6-S2-S7

**Published:** 2012-12-12

**Authors:** Shingo Kikugawa, Kensaku Nishikata, Katsuhiko Murakami, Yoshiharu Sato, Mami Suzuki, Md Altaf-Ul-Amin, Shigehiko Kanaya, Tadashi Imanishi

**Affiliations:** 1 Integrated Databases and Systems Biology Team, Biological Information Research Center, National Institute of Advanced Industrial Science and Technology (AIST), Tokyo, Japan; 2 Department of Bioinformatics and Genomics, Graduate School of Information Science, Nara Institute of Science and Technology (NAIST), Japan; 3 VALWAY Technology Center, NEC Soft Ltd, Japan

## Abstract

**Background:**

Proteins interact with other proteins or biomolecules in complexes to perform cellular functions. Existing protein-protein interaction (PPI) databases and protein complex databases for human proteins are not organized to provide protein complex information or facilitate the discovery of novel subunits. Data integration of PPIs focused specifically on protein complexes, subunits, and their functions. Predicted candidate complexes or subunits are also important for experimental biologists.

**Description:**

Based on integrated PPI data and literature, we have developed a human protein complex database with a complex quality index (PCDq), which includes both known and predicted complexes and subunits. We integrated six PPI data (BIND, DIP, MINT, HPRD, IntAct, and GNP_Y2H), and predicted human protein complexes by finding densely connected regions in the PPI networks. They were curated with the literature so that missing proteins were complemented and some complexes were merged, resulting in 1,264 complexes comprising 9,268 proteins with 32,198 PPIs. The evidence level of each subunit was assigned as a categorical variable. This indicated whether it was a known subunit, and a specific function was inferable from sequence or network analysis. To summarize the categories of all the subunits in a complex, we devised a complex quality index (CQI) and assigned it to each complex. We examined the proportion of consistency of Gene Ontology (GO) terms among protein subunits of a complex. Next, we compared the expression profiles of the corresponding genes and found that many proteins in larger complexes tend to be expressed cooperatively at the transcript level. The proportion of duplicated genes in a complex was evaluated. Finally, we identified 78 hypothetical proteins that were annotated as subunits of 82 complexes, which included known complexes. Of these hypothetical proteins, after our prediction had been made, four were reported to be actual subunits of the assigned protein complexes.

**Conclusions:**

We constructed a new protein complex database PCDq including both predicted and curated human protein complexes. CQI is a useful source of experimentally confirmed information about protein complexes and subunits. The predicted protein complexes can provide functional clues about hypothetical proteins. PCDq is freely available at http://h-invitational.jp/hinv/pcdq/.

## Background

Proteins interact with other proteins or biomolecules to perform their functions, and protein complexes are the fundamental functional units of these macromolecular systems. Comprehensive analysis of PPIs provides a valuable framework for understanding the protein functions required for various biological processes in cells. Moreover, it can provide annotation clues about proteins with unknown function [1-3].

An important issue for the elucidation of the functional organization of the proteome is the extraction of information about protein complex formation and function from the PPI network.

In recent years, a number of well-organized public PPI databases have become available, including Biomolecular Interaction Network Database (BIND) [4,5], Database of Interacting Proteins (DIP) [6], Molecular INTeraction database (MINT) [7,8], Human Protein Reference Database (HPRD) [9], IntAct [10], and Genome Network Project Y2H data (GNP-Y2H; http://genomenetwork.nig.ac.jp/index_e.html, NOT http://genomenetwork.nig.ac.jp/ ). In the present PPI data, the main focuses are on protein-binding partners or binary protein interactions. Knowledge about how gene products form complexes, interactions among complexes, or protein interconnectivity in a complex is still scarce. The overlap of PPI data entities across databases is relatively low. The existence of only a partial map of the whole interactome space limits the broad application of systems modeling. Accordingly, it is essential to integrate PPI data in order to fill in as many holes in the interactome space as possible. Some integration of the above PPI data has been conducted by STRING [11], OPHID [12], and HAPPI [13]. However, protein complex information has been poorly annotated in these resources.

Several human protein complex databases have been developed to date, including CORUM [14,15] and disease-related complex [16]. The protein complexes in CORUM were collected only from literature. The database does not provide information about many uncharacterized proteins whose interactions are supported by PPI data. The disease-related complex database [16] is focused on disease complexes, using information on proteins known to be involved in similar disorders. Accordingly, it contains a relatively small number of complexes (506) and lacks many other important complexes.

In this study, we integrated human PPI data from the six databases and predicted human protein complexes from the integrated PPI data set by finding densely connected regions with cluster properties in the PPI network based on graph theory as described in our previous report [17]. The novelty of prediction methods is that we optimized parameter settings for the prediction tool DBClus using an original correct dataset. After prediction, experienced annotators manually annotated the predicted protein complexes according to our standardized procedures, using literature mining and the wealth of annotation data in the human full-length cDNA database "H-Invitational Database" (H-InvDB) that we developed [18-20]. Using the data from H-InvDB, we performed several analyses of the annotated complexes to increase the validity of our annotation. This is the first attempt at comprehensive manual curation of human protein complexes predicted from PPI networks.

## Construction and content

### Integration of PPI data into H-InvDB proteins

**Figure 1:**
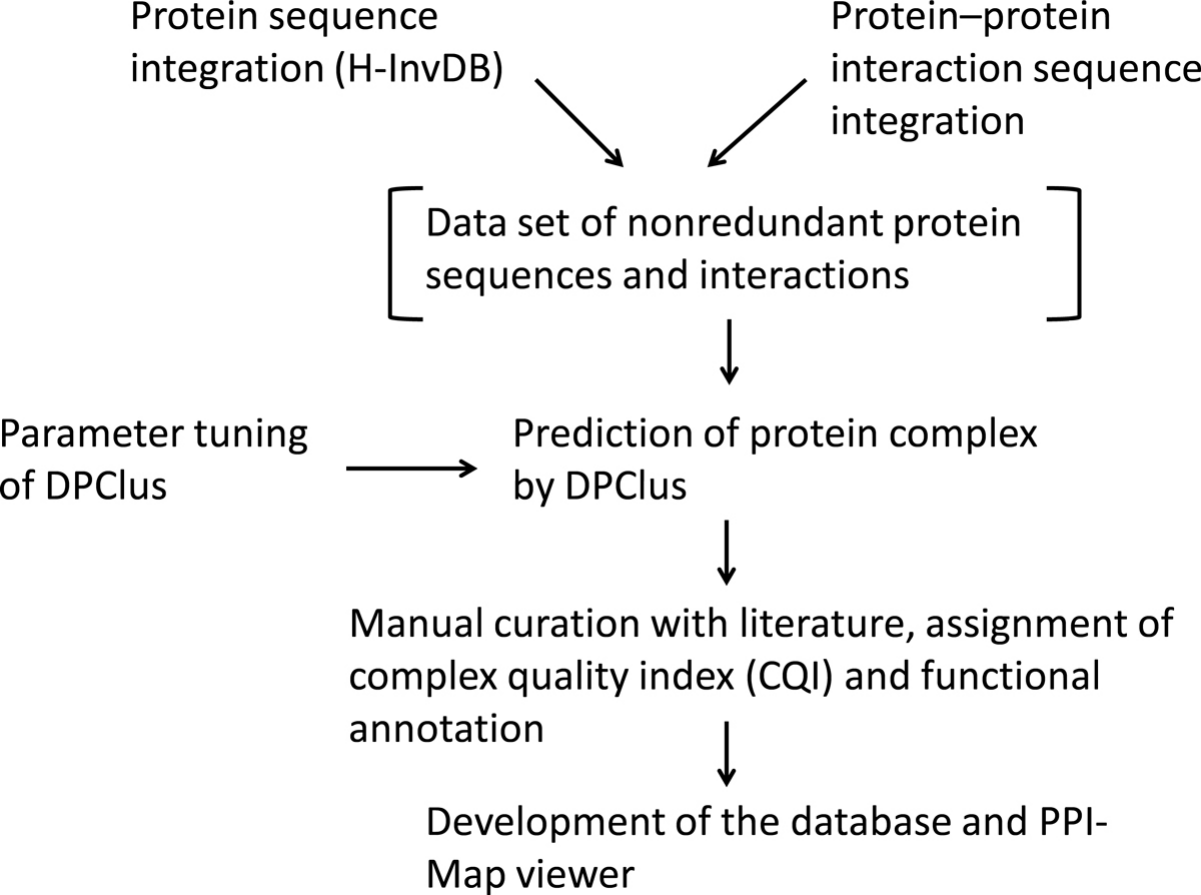
**A flowchart of the database construction process**.

The construction processes of the database are shown in Figure 1. It begins with two kinds of integration: protein sequences and PPI data sets. We have previously performed the integration of human protein sequences in the course of developing a comprehensive database of human genes and transcripts called H-InvDB (http://www.h-invitational.jp/) [18-20]. It is a unique database that integrates into a single entity the annotation of sequences, structure, function, expression, subcellular localization, evolution, and the diversity of human genes and their encoded proteins. It is useful as a platform for conducting *in silico *data mining. Our international collaboration for analysis of high-quality full-length cDNA clones, in addition to EST assemblies and CAGE tags, has resulted in the integrative annotation of 187,156 transcripts placed at 36,073 loci. Based on the open reading frame (ORF) prediction of H-InvDB transcript sequences, followed by the functional annotation of experienced annotators, we identified 108,530 nonredundant human protein candidates. We downloaded all protein sequences from GenBank [21], RefSeq [22], and UniProt [23] databases by their accession numbers and removed redundancies using BLASTCLUST [24,25] with a threshold of 98% sequence similarity in 95% alignment length coverage for both sequences. The resulting nonredundant sequences were named as "H-InvDB proteins" (Release 5.0).

To integrate PPI information, we collected PPI data from the six databases, BIND [4,5]; DIP [6]; MINT [7,8]; HPRD [9]; IntAct [10]; and GNP, as major resources for PPI. We used XML and flat files from PPI databases; BIND, DIP, MINT, HPRD, IntAct, and GNP on October 25, 2007. These databases, except for GNP, store experimentally determined PPIs from many organisms collected by literature curation, whereas GNP stores original Y2H experimental data on humans. Computationally predicted PPIs were excluded from this study. A standardized interaction data model is needed for storing PPI data from different sources. Following the method described in the Atlas biological data warehouse [26], we designed data loading applications for each PPI database and a relational data storage system compliant with the Proteomics Standards Initiative Molecular Interaction Standard (PSI-MI) controlled vocabulary [10], a community-standard XML format for the presentation of protein interaction data. This system allowed us to unify data from different sources. We used only human PPIs in this study and did not use cross-species PPI data such as human proteins interacting with mouse proteins or data with ambiguous taxonomic labels such as "Mammalia," commonly found in the HPRD download file. To survey human PPIs from the landscape of the human interactome, we mapped the PPI information onto the H-InvDB proteins. We removed PPI data redundancies by evaluating sequence similarity and then integrated human PPIs with the H-InvDB proteins. As a result, we obtained 32,198 human PPIs composed of 9,268 proteins.

**Figure 2:**
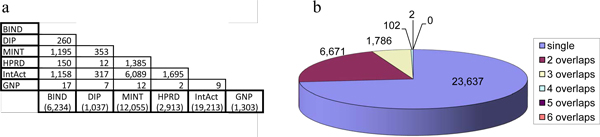
**Overlap of human PPIs in six PPI databases**. (a) Pairwise overlaps of PPIs across databases are shown in cells. The number of nonredundant PPIs is shown in parentheses for each database. (b) Overlaps of PPIs shared in common in one, two, three, four, five, and six databases are shown.

Figure 2 shows the overlap of human PPIs across the six databases. There are 6,234 nonredundant human PPIs in BIND whereas DIP; MINT; HPRD; IntAct; and GNP contain 1,037; 12,055; 2,913; 19,213; and 1,303 PPIs, respectively. Figure 2a shows pairwise overlaps of PPIs across the databases; MINT and IntAct share 6,089 PPIs, which is the highest overlap among these databases. As shown inFigure 2b, 6,671; 1,786; 102; and two PPIs are shared in 2; 3; 4; and 5 databases, respectively, but there are no PPIs in common among all the six databases. There are 23,637 unique PPIs in the databases, representing 73% of the PPI dataset. The overlap across these databases was relatively small, reflecting a much larger human interactome space than that represented by the currently known PPIs [27-29]. Thus, it is essential to integrate the PPI data to achieve a complete view of the human interactome.

### Prediction of protein complexes with clustering tool DPClus after parameter optimization using an original reference protein complex set

In a PPI network, nodes represent proteins and edges represent interactions. We previously developed an algorithm called DPClus, which extracted densely connected regions in a network and demonstrated that many of these regions correspond to known protein complexes or protein functional units [17,30]. DPClus is a robust algorithm unaffected by a high rate of false positives in data from high-throughput interaction-detection techniques [17]. DPClus can detect clusters of networks that are separated by sparse regions, keeping track of the periphery of a cluster by monitoring cluster properties of its neighbor. Thus the program considers two parameters, "network density" and "cluster property."

To evaluate the optimal values of these two parameters for predicting protein complexes, we used a set of experimentally determined protein complexes (the reference protein complex set). We manually collected 89 protein complexes from the scientific literature and retrieved 55 complexes from three-dimensional structures of human protein complexes recorded in the PDB [31]. We performed parameter optimization to select the two best parameters to achieve the best match of the predicted set with the reference complex set. DPClus was run many times for all possible combinations of the two parameters (network density and cluster property, varied from 0.0 to 1.0 with increments of 0.1). In the parameter optimization process, DPClus was restricted to finding complex sizes of three or more. For this case, a predicted complex needs at least two proteins in common with a known complex to be considered a match. Two scores were checked for each parameter set: the sum of recalls, which is a ratio of the number of matched proteins of a known complex to those of a predicted complex, and the sum of precisions, which is a ratio of the number of matched proteins of a predicted complex to those of a known complex. Recall and precision were zero when proteins of a known complex matched fewer than two proteins of a predicted complex. Recall and precision were one when proteins of a known complex matched perfectly to the proteins of a predicted complex. To avoid overprediction of duplicated complexes, which shared several proteins and matched an identical known complex, the best recall and precision scores were divided by their frequencies. For the best prediction performance of DPClus, the two parameters, network density and cluster property, were optimized using the largest protein subunits of the reference complex set. We simulated prediction with 100 different parameter sets and the best, with network density 0.6 and cluster property 0.5, was determined from the best ROC curves. With this parameter set, DPClus predicted 1,264 complexes matching 92 of the 144 known complexes. The average recall and precision of these 92 matched complexes were 0.54 and 0.66, respectively. We also calculated the average number of complexes that share a common protein. On an average, each protein was present in 1.24 complexes of the reference complex set. Using the optimized parameters gave a result identical to that for the predicted set. With this parameter set (network density 0.6, cluster property 0.5), we predicted 1,319 protein complexes in the integrated PPI network composed of 32,198 human PPIs.

In prediction of protein complexes by DPClus, we adopted the "overlapping clustering mode," which allows identical proteins to be classified into different clusters, because it is biologically well established that proteins can be present in multiple complexes at different times and locations. For example, POLR2E/RPB5 (HIP000039507), POLR2F/RPB6 (HIP000096671), POLR2H/RPB8 (HIP000027404), POLR2K/RPB12 (HIP000043404), and POLR2L/RPB10 (HIP000064404) are conserved throughout RNA polymerases I, II, and III [32]. Before complex prediction, we evaluated the optimal values of DPClus parameters by comparing the predicted complex set with the experimentally determined set of 144 reference complexes.

### Manual annotation of the predicted protein complexes: re-clustering, functional annotation, protein category, complex quality index (CQI), and naming of complexes

Using the clusters or protein complexes predicted by DPClus, we performed manual annotation by the following procedures: 1) curators searched the scientific literature for evidence that the proteins of the predicted complexes are experimentally defined complex members or subunits, 2) missing proteins were manually added to the predicted complexes if literature evidence showed that they were subunits of the complexes, and 3) data such as complex names; descriptions; localizations; and complex-complex interactions (CCIs), and their subunit functions, structures, expression profiles, gene loci, and PPIs among protein subunits were integrated. We did not exclude proteins that were predicted to be subunits but lacked literature evidence, instead considered them as complex subunit candidates. The provision of predicted candidates is one of the advantages of PCDq.

We assigned the protein subunits, or member proteins of complexes, of the predicted complexes to three categories based on the evidence levels: category I, proteins that are confirmed as subunits of known complexes in the literature or as ternary structures in the PDB [31]; category II, proteins for which no evidence of complex membership were found in the literature, but which have functions related to those of the shared category I subunits in the predicted complexes according to their protein definitions or Gene Ontology (GO) terms [33]; and category III, proteins that are predicted as complex subunits by DPClus and do not fall into the other two categories. Because our protein complex prediction allowed the same proteins to be subunits of different complexes, such shared proteins could be classified into different categories in different complexes.

To summarize the categories of all the subunits in a complex, we devised a CQI and assigned a CQI value to each complex. CQI is an index of the different levels of evidence for an annotated complex based on the protein category, defined by "[Number of category I proteins].[category II proteins].[category III proteins]/[Total number of proteins in a predicted complex]." For example, if the CQI of a complex is "5.2.1/8," the complex has eight subunits with five, two, and one protein classified into categories I, II, and III, respectively.

The predicted complexes were named based on scientific names from the literature, if the majority of proteins in a complex were common to a known complex and a name (e.g., exosome, spliceosome) for the complex was available; however, we used artificial descriptions using concatenated gene symbols when not all symbols of proteins were available (e.g., GLI1-STK36-SUFU complex, DBNL-ITK-PLCG1-SH3BP2 containing complex). Descriptions of complexes were quoted from references with their PubMed IDs. Functional categories and subcellular localizations were added if the descriptions were available in the literature.

### Database of protein complex annotations and visualization tool PPI-Map for CCIs

**Figure 3:**
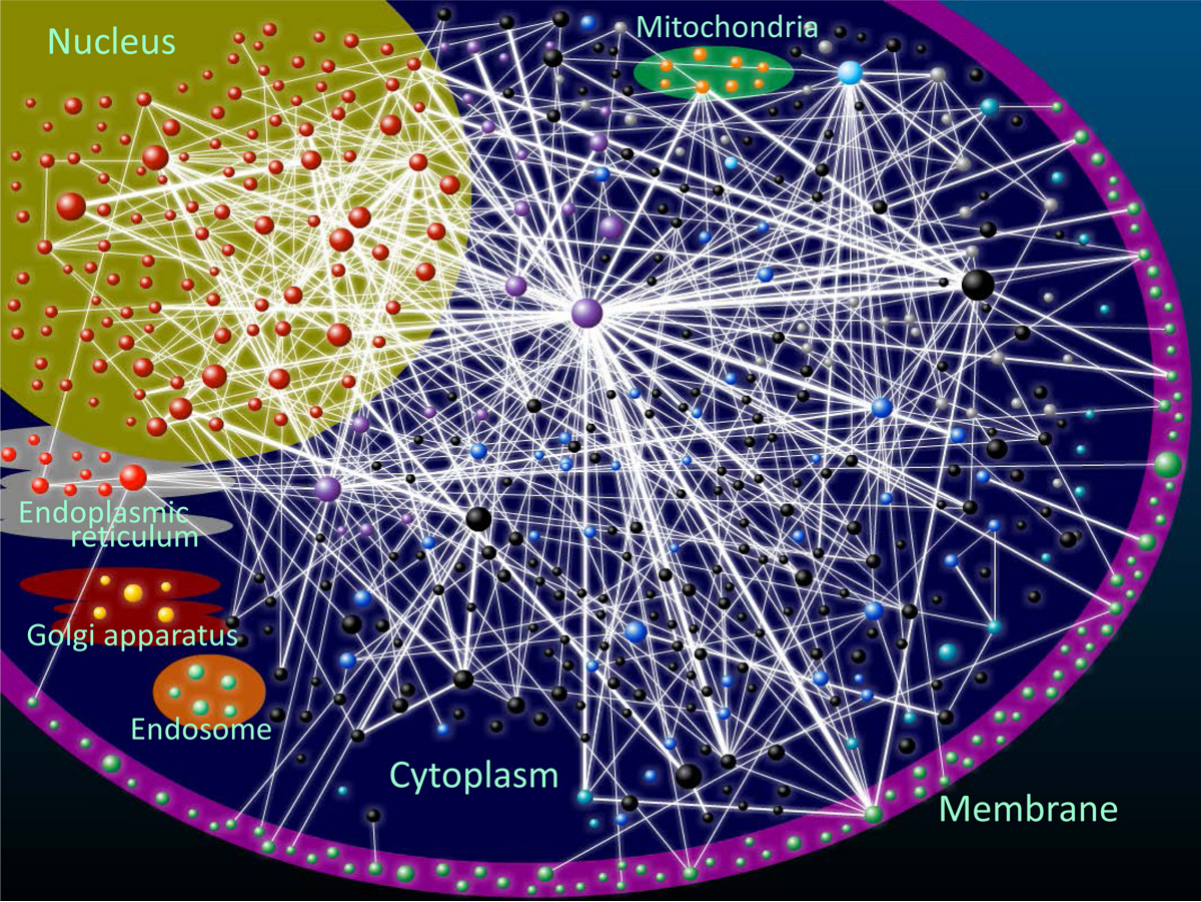
**A view of CCIs with the subcellar localizations of the annotated complexes**. Each node represents a complex and edges represent interactions. Node size represents the number of proteins in a complex and the thickness of edges connecting complexes, which are exponential to the number of PPIs between connected nodes. Node colors indicate subcellular localization of the annotated complexes; dark red: nucleus, blue: cytoplasm, green: membrane, purple: nucleus and cytoplasm, yellow: Golgi apparatus, blue-green: cytoplasm and membrane, light blue: cytoplasm, membrane and nucleus, orange: mitochondria, light red: endoplasmic reticulum, light green: endosome, gray: other subcellular localization, black: NA/unknown.

The visualization tool PPI-Map in PCDq can show protein interconnectivity of a complex, complex-external protein interactions, and CCIs. To the best of our knowledge, PPI view is the first database that can show CCIs in the human interactome with detailed annotation. As shown in Figure 3Figure 3, using PPI-Map we have constructed a view of CCIs showing the subcellar localizations of the annotated complexes. In Figure 3, each node (circle) represents an individual complex and each edge represents an interaction. To avoid unnecessary complexity of the CCI network, 541 perfectly or partially matched complexes and interactions comprising more than 10 PPIs are shown. PPI-Map can be used to view CCIs graphically with the ability to scale seamlessly and to move and change the thickness of edges connecting complexes. Users can edit (delete, move, expand, etc.,) nodes and edges of the network.

The novel human protein complex database, called PCDq, provides three main views: protein complex information in the "protein complex view," integrative overview of a PPI in the "PPI view," and network information including both PPI and CCIs in "PPI-Map." The complex view provides names, functions, protein subunits, subunit roles, and CQL. PPI view provides PPI partners for a specified protein. Finally the new visualization tool PPI-Map allows users to visualize protein interactions graphically: not only PPIs among the protein subunits but also CCIs, via a seamless and detailed annotation of each protein complex and its subunits. These three views have hyperlinks to one other and also to transcript/locus/protein views of the H-InvDB human gene/transcript/protein database. Considering all of these features, PCDq is a useful platform for understanding protein function from the viewpoint of protein complexes as another important functional level, as well as their interactions. The CQI provides unique and reliable clues for inferring some roles of proteins whose functions are unknown.

### Statistics of PCDq

**Figure 4:**
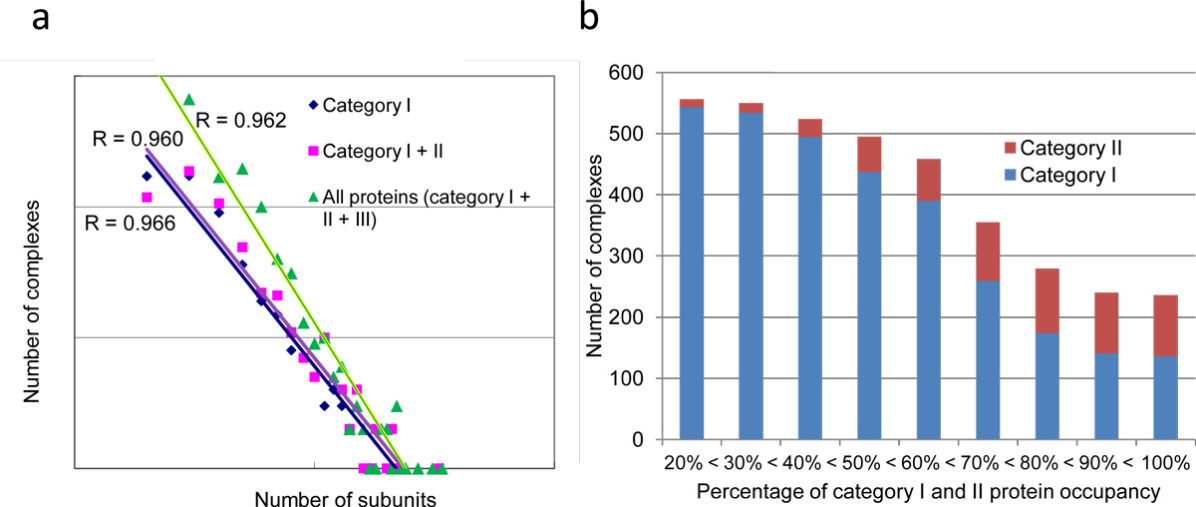
**Relationship between complexes and subunits**. (a) The relationship between complex size (number of different protein subunits of each category; X-axis) and frequency (Y-axis). (b) Percentage of category I and II protein occupancy of the annotated complexes.

In total, we predicted and annotated 1,264 protein complexes. A list of all annotated complexes is available at the PCDq site. Category I contained 2,106, category II 299, and category III 3,273 proteins, with protein subunit sharing allowed (Table 1a). The average number of proteins per complex was slightly different among the categories: 3.9 for category I proteins only, 4.3 for proteins in category I and II, and 4.5 for proteins in all the three categories. However, the size distribution in the datasets was quite diverse. Figure 4a shows a plot of the number against the size (number of protein subunits) of complexes. The relationship follows an inverse power law.

**Table 1:** Protein and the complex annotation summary

Number of the proteins (a)	
H-InvDB proteins	108,530
Proteins in the PPI data set	9,268
Proteins in the predicted complexes	4,513
Category I	2,106
Category II	299
Category III	3,273

**Number of the complexes (b)**	

Perfectly matched	136
Partially matched	405
Hypothetical	723
Total	1,264

(a) Number of proteins in H-InvDB, the integrated PPI data set, and the predicted complexes. The categorized proteins in the predicted complexes are described in the text. Because complex sharing proteins could be classified into more than one category as subunits of different complexes, the total number of categorized proteins is greater than the number of nonredundant proteins in the predicted complexes. (b) Type of the predicted complexes. Three types of predicted complexes were defined by degree of matching to known complexes (details are in the text). Total number of the predicted complexes in this study is 1,264.

We defined three types of predicted complexes: perfectly matched, partially matched, and hypothetical complex. These correspond to a complex with all subunits in category I, a complex with at least two proteins in category I, and a complex with all subunits in category III, respectively (Table 1b). By this annotation, the number of complexes was 136 for type I, 405 for type II, and 723 for type III Table 1b).

**Figure 5:**
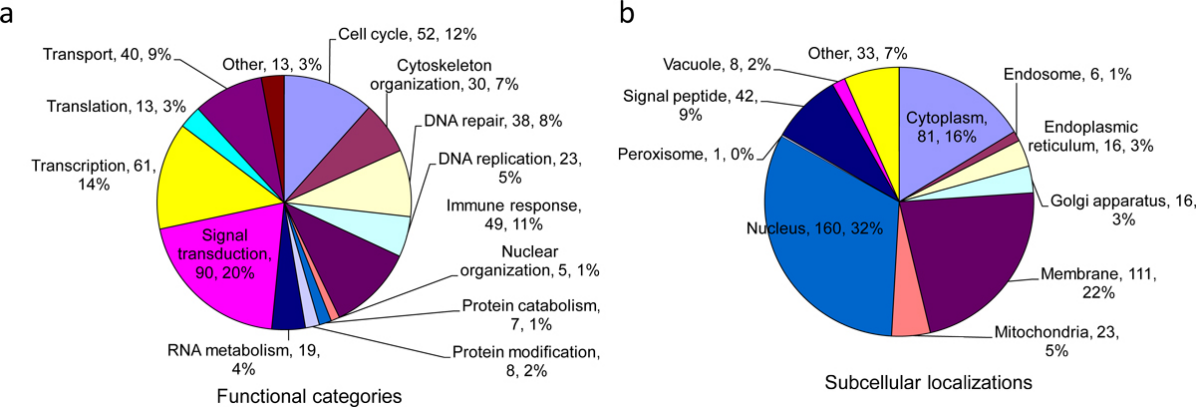
**Protein complex profiles**. (a) Distributions of functional categories of the annotated complexes. (b) Distribution of subcellular localizations of the annotated complexes.

From information in the literature, we assigned functional categories and subcellular localization to the annotated complexes (Figure 5a,b). The major functional categories were signal transduction (90 complexes, 19%), transcription (61, 14%), cell cycle (52, 12%), and immune response (49, 11%). More than 70% of the complexes are localized in the cell nucleus (160, 33%), membranes (111, 22%), and cytoplasm (81, 16%).

### Consistency of GO terms assigned to subunits in a complex

Given that proteins in a complex cooperatively play a biological role, it is expected that they are present in the same location in a cell at a certain time and that they act cooperatively in the same biological process or pathway. To assess the quality of our protein complex annotation, we calculated the enrichment ratio of consistency of GO terms among subunits of a complex. This assessment is based on the assumption that the same GO terms are assigned to proteins in a single protein complex.

All GO terms of "biological process," "cellular component," and "molecular function" assigned to the H-InvDB transcripts were used for this study. The depth of GO terms from the root in the GO hierarchy was set to five and GO terms representing nodes with depth less than five were ignored. If the GO term assigned to the transcript had depth greater than five, the corresponding parental node with depth five was reassigned and redundancy was removed. As a control set representing the entire proteome, we collected GO terms assigned to all 36,073 representative transcripts in H-InvDB. All protein subunits in 1,264 complexes were used as one set of protein complexes (PCset1) for the assessment. To construct the manually curated set of protein complexes (PCset2), we collected only category I proteins from perfectly or partially matched complexes (these complexes were defined in the subsection "Statistics of PCDq") and discarded category II or III proteins, which have not been described as subunits of a complex in the literature. PCset2 contained 541 complexes.

First, we estimated the enrichment of some GO terms in a complex compared to GO terms assigned to the proteome. The proteome set comprised 36,073 proteins, each derived from a distinct locus or gene of H-InvDB. The enrichment of GO terms was examined against two sets of protein complexes, PCset1 and PCset2. Significance of enrichment of a given GO term in a complex was tested by one-sided Fisher exact test for a 2 × 2 contingency table (A, B, C, D). "A" represents the number of subunits expressing the given GO term, and "B" is the number of subunits not having the GO term in the protein complex. "C" and "D" represent the corresponding numbers estimated for the entire proteome.

To estimate the quality of protein complex annotation, we defined another quality index, the "GO consistency index." This index for a given protein complex is estimated by the following equation:

$$\text{GO}\;\text{consistency}\;\text{index}={\text{N}}_{\text{cons}}\text{/}{\text{N}}_{\text{all}},$$

where N_cons _is the number of edges that connect two proteins sharing the same GO term and N_all _is the number of possible combinations (edges) for all subunits of the complex.

It was observed that 450 of 1,264 PCset1 (35.6%) protein complexes had one or more enriched GO term (Fisher exact test, p-value ≤ 0.01). In contrast, 254 of the 541 PCset2 complexes (47%) had one or more enriched GO term. The ratio of protein complexes having enriched GO terms was greater in PCset2 than in PCset1, suggesting that the reliability of protein complex annotation was refined by manual checking.

**Figure 6:**
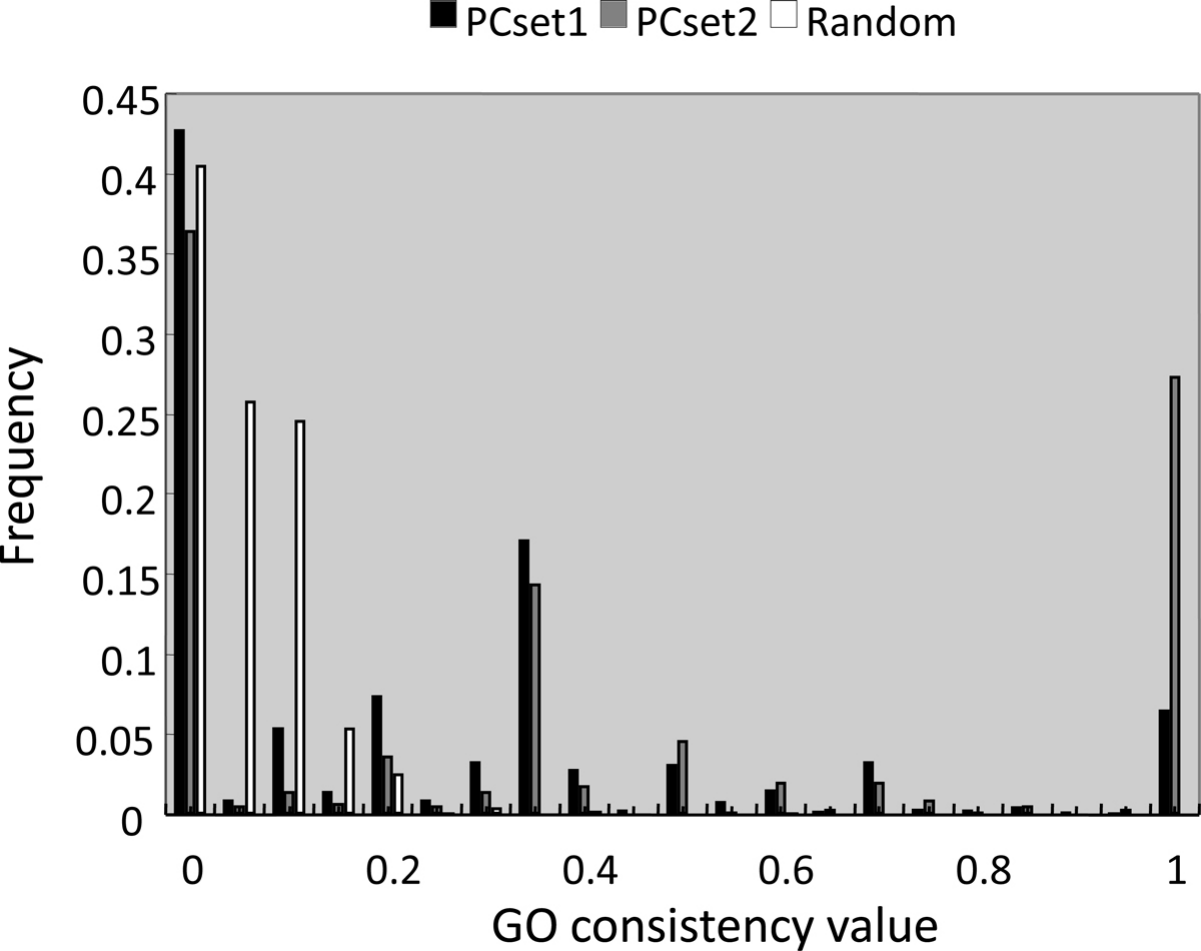
**Distributions of GO consistency index in PCset1, PCset2, and random set**. Histogram of GO consistency index for protein complexes in PCset1, PCset2, and random set shows a shift toward larger values in the PCset1 and PCset2 than in the random set.

The degree of consistency of GO terms among subunits in a complex was estimated; i.e., the homogeneity of GO terms assigned to complex subunits. A consistency index (see Materials and Methods) was used as an indicator of homogeneity. With the object of estimating the degree of GO term consistency expected by chance, 100 sets of randomly selected genes from H-InvDB, all representative transcripts with complex sizes matching our annotation of PCset1, were created and used as a control. Average consistency indexes were estimated to be 0.23, 0.41, and 0.04 for protein complexes of PCset1, PCset2, and the random set, respectively. The value is higher in PCset1 (Student *t *test, p-value 2.9E-111) than in the random set, and in PCset2 than in PCset1 (p-value 1.6E-25). These results are still statistically significant after Bonferroni multiple-testing adjustment, which is relatively conservative. The histogram of consistency indexes for the three sets is shown in Figure 6. In particular, cases in which the consistency index was 1.0 (i.e., all subunits shared common GO terms with other subunits), increased dramatically after manual curation, indicating the relatively high quality of manual annotation and the advantage of protein complex prediction followed by manual annotation as opposed to only single computational prediction.

Intriguingly, we found 28 PCset1 unique complexes with consistency index 1.0. Although the existence of the protein complexes has not yet been validated experimentally, the compatibility between the prediction of protein complexes by our clustering method and the consistency of GO terms offers reliable candidates for novel functional protein complexes to be validated by future experiments.

### Similarity of gene expression profiles among proteins in the same complexes

Based on the idea that coexpressed genes are more likely to have the same or similar functions, cluster analysis of gene expression data has been used to predict the functions of non-annotated proteins [34,35]. Reversing the process, we examined whether proteins in the same complex (involved in the same functions) have similar expression profiles. For each complex, we compared the expression profiles of protein subunits in the complex. When the subunits of a complex are similar in their expression profiles, the profile should provide some functional information about a complex whose function is unknown.

Expression profiles of 729 complexes were obtained from the Human Anatomic Gene Expression Library (H-ANGEL) [36], the satellite database of H-InvDB. From the download file of H-ANGEL ("H-ANGEL_matrix.txt," December 2007 version), gene expression data measured by the iAFLP method [37] for 10 tissue categories were extracted. For some loci, multiple iAFLP-tags correspond to the same locus. In such cases, the different expression profiles for a single locus were averaged over the tags. The expression profile of a gene was expressed by a vector of 10 elements. The similarity of gene expression profiles between two loci was calculated as the cosine of the two vectors. The similarity of multiple gene expression profiles for subunits of a protein complex was defined by the averaged cosines of all combinations of all the different subunits. The cosines of a complex were evaluated by simulation. For every number (*k*) of subunits in the complex, we randomly selected *k*-genes from genes having expression profiles. We then calculated the averages of the cosines of the expression profiles. We repeated the procedure 100,000 times for every number of subunits (*k*), and used the results for p-value estimation.

Of 729 complexes, seven were found to have significant gene expression similarity by a false discovery rate (FDR) criterion of 0.05. FDR, the expected proportion of incorrectly rejected null hypotheses, is a widely used statistic for multiple testing [38]. The seven complexes are shown in Table 2.

**Table 2:** Protein complexes comprising protein subunits with significantly similar gene expression profiles

**Complex No**.	CQI	Complex name	cosine	FDR	# of genes
30	21.1.0/22	19S proteasome of the 26S proteasome	0.92	0.001	13
12	18.0.4/22	20S proteasome of the 26S proteasome	0.88	0.006	17
41	12.1.0/13	RNA polymerase II complex	0.92	0.008	10
68	0.0.11/11	COP9 signalosome (CSN)	0.92	0.014	9
953	0.0.3/3	GAGE6-GMCL1L containing complex	1.00	0.022	3
130	3.0.8/11	Fibrinogen	0.96	0.037	4
77	4.0.8/12	18S U11/U12 complex	0.89	0.041	14

**Figure 7:**
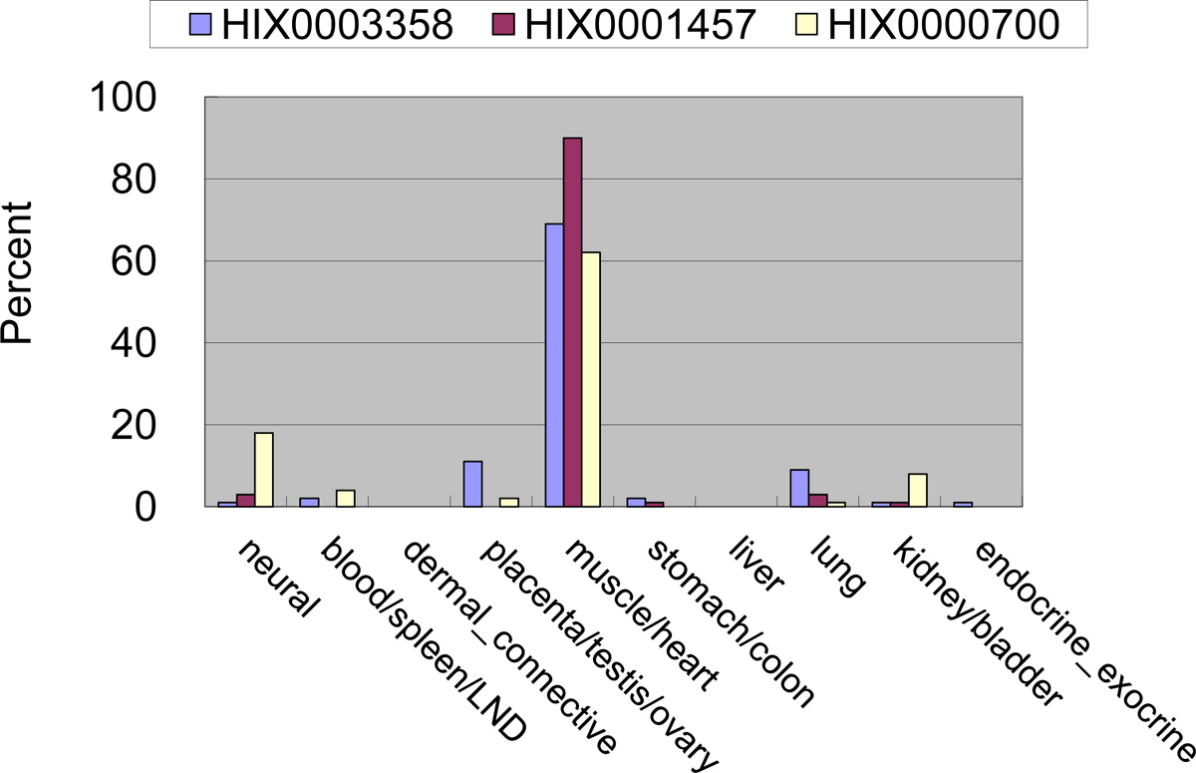
**Relative percentage of gene expression levels of the troponin complex**. The three gene loci of the troponin complex (complex 258) subunit proteins are expressed specifically in muscle/heart tissue.

Some of the most interesting complexes are those in which the expression of the protein subunits is similar and tissue specific. We found several such complexes using entropy of gene expression profile. Among these complexes, the fibrinogen complex (complex 130; liver specific, average entropy 1.20) was such a case. Other examples are the AK5-CPNE6-TRIM46 complex (complex 540) and the troponin complex (complex 258). Though the FDRs of the two complexes were not significant, 0.22 and 0.68, respectively, the gene expression profiles were very similar with cosines of 0.99 and 0.95, respectively. For troponin, the gene expression of the subunits is specific to that of muscle/heart tissue (average entropy 1.12). The gene expression profiles of the three subunits in troponin complex are shown in Figure 7. The similarity of these expression profiles suggests that they function as a complex.

**Figure 8:**
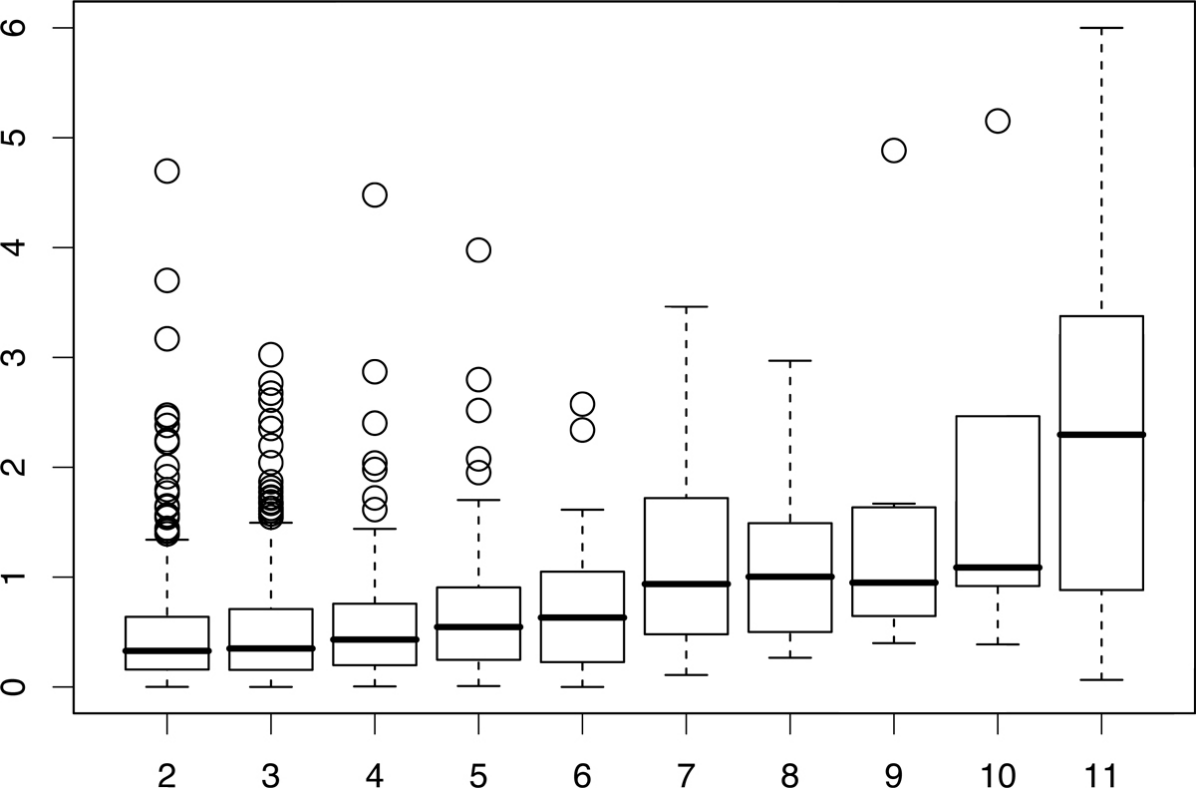
**Box plot of gene expression profile similarity and the number of protein subunits in a complex**. The y-axis indicates gene expression similarity (negative logarithm of p-value of average cosine of gene expression profiles) in a complex; a higher value means that the subunits of the complex show greater similarity in their gene expression profiles. The x-axis indicates the number of protein subunits with expression data in the complex. The gene expression profiles similarity increases with the number of proteins.

As shown above, the gene expression of the protein subunits was not significantly similar in most of the predicted protein complexes. However, we found that the gene expression of protein subunits is more likely to be similar for large complexes.We calculated the p-values of gene expression similarities for each complex and plotted the distribution of p-values for different numbers of proteins in a complex (Figure 8). The figure illustrates that similarity in gene expression of proteins in the same complex increases as the number of protein subunits (complex size) increases. This is the first report of a relationship between expression similarity and complex size in human PPI and is consistent with results reported for yeast [39].

### Relationship between the establishment of protein complexes and gene duplication

To investigate the contribution of gene duplication to the establishment of protein complexes, we examined portions of duplicated genes (proteins) or paralogs in the complexes.

For all combinations of subunits in a protein complex, we evaluated whether the genes were paralogous (two genes copied by segmental duplication) following the method of Gu et al. [40]. Gene models that were mapped onto "random" or "haplotype" contigs were not used in the analysis. FASTA package version 34t25 [41] was used for the analysis. In addition, we conducted another paralog analysis with BLASTP using less stringent criteria for the assignment of duplicated genes. BLAST version 2.2.17 was used. If the gene pair showed similarity with E-value less than 1E-05, we assigned it as paralogous.

This paralog assignment method yielded 2,353 duplicated genes in a total of 4,191 genes that were the components of 1,264 complexes. Of the 1,264 complexes, 336 (26.5%) were judged to have at least one paralog pair. Moreover, we obtained 218 complexes (17.2%) in which more than half of the components were judged to be paralogous to another gene in the same complex. Using a less stringent method with BLASTP (E-value ≤ 1E-05), these percentages were estimated to be 38.5% and 27.3%, respectively.

The replication factor C (RFC) complex (complex 105) is a good example of the formation of a protein complex induced by gene duplication. This complex consists of five RFC subunits and one binding partner, PCNA [42]. The complex is known to be associated with DNA synthesis [42], and the function and machinery are conserved between yeast and human [43], indicating that this is an ancient protein complex. Paralog assignment suggested that three (RFC 36, 37, 40) of five RFC subunits are paralogous; i.e., originating from a common ancestor, whereas the result obtained by the less stringent BLASTP method suggested that all five subunits are mutually paralogous. The presence of the "RFC box" motif in all five proteins and the consistency of exon-intron boundaries also support the homologous relationships of these five subunits. These results indicate that the enlargement of a protein complex is mainly mediated by homologous interactions and that gene duplication events markedly contribute to the establishment of protein complexes.

### Functional assignments for hypothetical proteins in the annotated complexes

An important goal of proteomics is functional assignment for proteins that cannot be annotated by homology alone. Several approaches for functional assignment from PPIs have been developed [1-3].

First, we explain the definition of proteins with no functional assignments, known as "hypothetical proteins." H-InvDB proteins were analyzed with standardized functional annotation by curators who classified the proteins into several categories: i) identical to known human proteins, ii) similar to known proteins (having 50% sequence similarity), iii) interPro-domain-containing proteins, and iv) hypothetical proteins (with no biological functions inferred). The "hypothetical proteins" discussed here are of the fourth category.

Next, we explain how the functions of those hypothetical proteins can be inferred. In PDBq we found 78 hypothetical proteins (as defined in H-InvDB) in the 82 predicted complexes. Although the majority (61 proteins, 78.2%) were subunits of 67 hypothetical complexes (none of their subunits were reported as complexes in the literature), 13 hypothetical proteins were subunits of 12 complexes whose functions were strongly deduced because at least half of their subunits were annotated as common to known complexes. A protein complex is thought to be a functional unit in which proteins combine to perform biological functions; accordingly, a hypothetical protein can be assigned a function related to that of the complex it joins. For example, two hypothetical proteins HIP000013164 and HIP000053526 were in the "dREAM complex" (complex 24), which is tightly bound to E2F-regulated promoters in G0 and dissociates from these promoters in the S phase of the cell cycle. In addition, some subunits of the complex can also interact specifically with MYB and may be involved in expression of MYB-dependent genes important in G2/M progression [44]. We expected that these two hypothetical proteins would then join the dREAM complex and might play a role in the cell cycle. Moreover, we found that annotated complexes such as the "Fanconi anemia (FA) core complex" (complex 61), "INO80 complex" (complex 75), and "Lamins complex" (complex 101) include hypothetical proteins (HIP000177716 for the FA core complex, HIP000079962 for the INO80 complex, and HIP000024165 for the Lamins complex). These complexes have DNA repair, DNA repair and transcription, and nuclear organization functions, respectively. Accordingly, these hypothetical proteins might also have functions associated with those complexes. Table 3 summarizes the 13 hypothetical proteins and 12 complexes, including hypothetical proteins as subunits and at least half of whose subunits are common to known complexes and their CQIs.

**Table 3:** Hypothetical proteins whose functions can be easily inferred from their partners

HIP (protein ID)	**Complex No**.	CQI	Name	Confirmed later
HIP000013164	24	10.1.2/13	dREAM complex	yes
HIP000053526	24	10.1.2/13	dREAM complex	yes
HIP000177716	61	8.0.1/9	Fanconi anemia (FA) core complex	yes
HIP000079962	75	11.0.2/13	INO80 complex	yes
HIP000024165	101	4.0.1/5	Lamins complex	no
HIP000046613	200	3.0.2/5	C8orf32-EFCBP2-RUNX1T1-ZNF652 containing complex	no
HIP000038372	673	4.0.1/5	BCL2A1-BCL2L1-BCL2L2-HRK-PMAIP1 complex	no
HIP000089800	922	2.0.1/3	HIF-1alpha-pVHL-ElonginB-ElonginC complex	no
HIP000027799	940	3.0.1/4	SRGAP3-WASF1 containing complex	no
HIP000060581	960	3.0.2/5	C19orf25-KNTC1-ZW10 containing complex	no
HIP000015491	967	3.0.2/5	NONO-PSPC1-WBP4-ZNRD1 containing complex	no
HIP000114159	1156	2.0.2/4	NUTF2-RAN complex	no
HIP000091971	1310	4.0.3/7	SCF (Skp1, cullin 1, F-box) ubiquitin E3 ligase complex	no

Thirteen hypothetical proteins whose complexes have at least half category I subunits are shown.

After annotation, we found that some of the hypothetical proteins were reported in the literature as actual protein subunits (Table 3). The results show the high potential value of our predicted complex data and indicate that the complex annotation used for our database can be a key tool for new discovery of protein complexes and their functions.

## Utility

PCDq comprises both known and predicted complexes and subunits. The evidence level for each subunit was also determined and summarized as a complex quality index (CQI) for each protein complex.

The expected users of PCDq are both experimental biologists and computational scientists. Biologists can seek candidate protein subunits for known or unknown protein complexes and review the information (functions, gene expressions, PPIs, etc.) about a protein complex. Computational scientists can collect integrated PPI network datasets with various levels of reliability using original annotation in the form of protein categories and CQIs. Thus, for users who would like to develop a method for protein complex prediction, PCDq provides different thresholds for dataset assembly using CQI.

Users can download the dataset of PCDq, including protein complex list, their subunits (members), and related functional annotation from the H-InvDB download page (http://h-invitational.jp/hinv/dataset/download.cgi, see "Results of computational analysis").

## Discussion

To assess the quality of our protein complex annotation, we estimated the enrichment and the proportion of consistency of GO terms among subunits of a complex. This assessment is based on the assumption that the same GO terms are assigned to the proteins in a single protein complex. The proportions of protein complexes having enriched GO terms and the degree of GO term consistency were greater in the manually curated set of protein complexes (PCset2) than in all the predicted complexes (PCset1) or the random set, indicating the relatively high quality of manual annotation and the advantage of protein complex prediction followed by manual annotation as opposed to only single computational prediction.

Next, for each complex, we compared the expression profiles of the protein subunits in the complex based on the idea that proteins in the same complex would have similar functions and that coexpressed genes are more likely to have similar functions. The result showed that the subunits of large complexes tend to be expressed similarly. The ratio of duplicated genes to all the proteins in a complex was evaluated, and the results indicated that the enlargement of a protein complex is mainly mediated by homologous interactions and that gene duplication events markedly contribute to the establishment of protein complexes.

Recent statistics of H-InvDB proteins show that 35% of H-InvDB representative transcripts are hypothetical proteins. Assigning functions to hypothetical proteins of unknown function is one of the most important issues in proteome analysis. Since subunits of a complex generally tend to have the same biological function, prediction of a protein complex allows increased confidence in the annotation of hypothetical proteins. After the construction of PCDq by protein complex prediction and annotation, we found that 78 hypothetical proteins were contained in the 82 predicted complexes. Of these 78, 13 were subunits of 12 functionally annotatable complexes. These hypothetical proteins are probably involved in biological processes shared by other subunits of their complexes. Thus complex prediction gives us some clues for inferring their functions. For example, it is suggested that the hypothetical proteins HIP000013164 and HIP000053526 in the dREAM complex function in the cell cycle, and that HIP000177716 (FA core complex), HIP000079962 (INO80 complex), and HIP000024165 (Lamins complex) function in DNA repair, DNA repair and transcription, and nuclear organization, respectively. The remaining eight hypothetical proteins that could be assigned functions are summarized in Table 3. In fact, when we checked the recent literature after making the predictions, four of the thirteen hypothetical proteins were found to be in fact subunits of the predicted protein complexes, and their PCDq entries were updated. Thus, protein complex prediction and annotation offers clues to the functions of hypothetical proteins.

## Conclusions

We predicted and annotated 1,264 human protein complexes from integrated PPI data. GO analysis increased the reliability of both complex prediction and manual annotation. The analysis of expression profiles and duplicated genes made it clear that protein subunits tend to be expressed similarly and are mutually paralogous within complexes. Comprehensive protein complex prediction and annotation will provide strong functional annotation clues about hypothetical proteins. We constructed a new human protein complex database with quality index (PCDq) to provide this comprehensive annotation of human protein complexes.

## Availability and requirements

PCDq is freely available at the URL http://h-invitational.jp/hinv/pcdq/.

## Abbreviations

BIND: (Biomolecular Interaction Network Database); BLAST: (Basic Local Alignment Search Tool); CAGE: (Cap Analysis of Gene Expression); CCI: (Complex-Complex Interaction); cDNA: (Complementary DNA); CQI: (Complex Quality Index); DIP: (Database of Interacting Proteins); EST: (Expressed Sequence Tag); FDR: (False Discovery Rate); GNP: (Genome Network Project); GO: (Gene Ontology); H-ANGEL: (Human Anatomic Gene Expression Library); H-InvDB: (H-Invitational Database); HPRD: (Human Protein Reference Database); iAFLP: (introduced Amplified Fragment Length Polymorphism); MINT: (Molecular INTeraction database); ORF: (Open Reading Frame); PCDq: (protein complex database with quality index); PDB: (Protein Data Bank); PPI: (Protein-Protein Interaction); PSI-MI: (Proteomics Standards Initiative Molecular Interaction Standard); RFC: (Replication Factor C); ROC: (Receiver Operating Characteristic); XML: (Extensible Markup Language).

## Competing interests

The authors declare that they have no competing financial interests.

## Authors' contributions

The first three authors contributed equally to this study. SK and KN initially designed the study, collected and integrated PPI data, predicted complexes by DPClus, developed the complex-annotation system PCDq and PPI-Map, and wrote the manuscript. KM joined designing of the study, analyzed gene expression profiles, arranged PCDq, updated data especially newly found functions of previously hypothetical proteins, and wrote the manuscript. YS was responsible for the analysis of GO terms consistency in complexes and the contribution of gene duplication to complexes. MS directed a collaborative activity for the manual annotation of complexes. AA and SK developed and improved DPClus. TI participated in its coordination and helped to draft the manuscript. All authors read and approved the final manuscript.
